# Deep Learning-Assisted Automatic Diagnosis of Anterior Cruciate Ligament Tear in Knee Magnetic Resonance Images

**DOI:** 10.3390/tomography10080094

**Published:** 2024-08-13

**Authors:** Xuanwei Wang, Yuanfeng Wu, Jiafeng Li, Yifan Li, Sanzhong Xu

**Affiliations:** 1 Department of Orthopedics, The First Affiliated Hospital, Zhejiang University School of Medicine, Hangzhou 310000, China; 1507130@zju.edu.cn (X.W.); lijiafeng@zju.edu.cn (J.L.); 2 Zhejiang Lab, Hangzhou 310000, China; yuanfeng_wu@outlook.com

**Keywords:** anterior cruciate ligament, deep learning, magnetic resonance imaging

## Abstract

Anterior cruciate ligament (ACL) tears are prevalent knee injures, particularly among active individuals. Accurate and timely diagnosis is essential for determining the optimal treatment strategy and assessing patient prognosis. Various previous studies have demonstrated the successful application of deep learning techniques in the field of medical image analysis. This study aimed to develop a deep learning model for detecting ACL tears in knee magnetic resonance Imaging (MRI) to enhance diagnostic accuracy and efficiency. The proposed model consists of three main modules: a Dual-Scale Data Augmentation module (DDA) to enrich the training data on both the spatial and layer scales; a selective group attention module (SG) to capture relationships across the layer, channel, and space scales; and a fusion module to explore the inter-relationships among various perspectives to achieve the final classification. To ensure a fair comparison, the study utilized a public dataset from MRNet, comprising knee MRI scans from 1250 exams, with a focus on three distinct views: axial, coronal, and sagittal. The experimental results demonstrate the superior performance of the proposed model, termed SGNET, in ACL tear detection compared with other comparison models, achieving an accuracy of 0.9250, a sensitivity of 0.9259, a specificity of 0.9242, and an AUC of 0.9747.

## 1. Introduction

Anterior cruciate ligament (ACL) tears are among the most common knee injuries worldwide, especially among young and active people. Severe tears require ACL reconstruction (ACLR) to restore stability, with more than 100,000 cases in the United States alone each year [1]. ACL can further lead to knee instability, which may progress to osteoarthritis and ultimately result in knee replacement surgery [2]. However, even with ACLR, more than 50% of patients still experience signs of osteoarthritis [3]. Therefore, the timely and accurate assessment of ACL tears is crucial for selecting the best treatment plan and effectively evaluating the patient prognosis [4]. It is of significant clinical importance for patients to regain normal knee stability and normal motor function, and to prevent or minimize secondary injuries to other knee structures [5]. Common methods for diagnosing ACL tears typically include clinical examinations, imaging studies, and arthroscopy [6]. Among these methods, arthroscopy is the gold standard for assessing internal knee joint diseases and other lesions [7]. However, it is relatively expensive and invasive [8]. Clinical tests, including the anterior drawer, Lachman, and pivot shift tests, are essential in post-injury assessments. When used together, these clinical tests have been shown to be highly specific for diagnosing ACL tears, but some experience is required to properly perform and interpret these tests [9]. Currently, Magnetic Resonance Imaging (MRI) is the best choice for identifying anterior cruciate ligament tears, but the accuracy of the results can vary, and it may depend on the level of experience of the reader, even when performed by musculoskeletal radiologists or sports orthopedic surgeons [10,11]. A previous study showed that MRIs of the ACL tears had an overall accuracy of 85.0%, a sensitivity of 82.5%, and a specificity of 92.8% [12]. Notably, the overall accuracy and specificity of ACL diagnosis can be improved with additional training each year, increasing the risk of misdiagnosis by inexperienced doctors [13]. For non-musculoskeletal radiologists, general orthopedic surgeons, or clinical doctors without a specialty in knee joint surgery, the accurate diagnosis of ACL tears may be challenging [14]. Therefore, making an accurate diagnosis of ACL tears through knee MRI remains challenging.

Automated systems utilizing deep learning can assist clinicians in reading knee MRI scans and formulating diagnoses [15]. In clinical diagnosis, detecting tears in the anterior cruciate ligament (ACL) relies on a composite assessment of oblique structures across multiple image slices with varying tissue contrasts, using MRI results that include fiber discontinuities, contour changes, and signal abnormalities within the injured ligament [16]. Deep learning models can automatically learn hierarchical feature representations from raw image data, capturing complex patterns and details that are difficult to design manually. This enables deep learning to effectively model subtle visual features present in medical images and complete the task of capturing subtle relationships in medical image interpretation [17]. Some studies have demonstrated the effectiveness and efficiency of deep learning methods in MRI analysis [18,19,20]. Investigating the capacity of deep learning methods for detecting ACL tears could establish whether such approaches are beneficial for diagnosing complex musculoskeletal abnormalities in MRI.

In natural image analysis, attention mechanisms have achieved widespread success and continue to captivate interest among researchers [21,22,23,24]. The attention method simulates human attention by assigning varying levels of importance to different parts of the input. The operation is conducted on low-level visual details or on high-level semantic contents. This helps the model discover the most relevant part of the query image. The application of attention mechanisms becomes particularly pertinent in medical image analysis because their structures are inherently intricate and the information is dense. The complexity inherent in medical images can potentially hinder diagnostic accuracy if not properly navigated. Attention can help the model concentrate more on the task-related region and extract a better feature representation, thereby enhancing analytical precision.

In this study, inspired by the success of deep learning-based methods in MRI analysis and attention mechanisms, we developed a deep learning model for detecting ACL tears in knee MRI examinations. The model consists of three main modules: a Dual-Scale Data Augmentation module (DDA), a selective group attention (SG) module, and a fusion module. The following are our work contributions:

(1) We established a deep learning-based method to detect ACL tears using MRI as an input.
(2) This study extends the augmentation strategy to both the spatial scale and layer scale, in order to address the challenge of limited data.
(3) The proposed method adopts a selective group attention module that examines the relationships among layers. A fusion module is used to integrate multiple perspectives, which simulates the clinical diagnosis process, to achieve the final classification.
(4) Several experiments were conducted to compare the proposed method and the baseline methods. The experimental results demonstrate the superiority of the proposed method and verify the effectiveness of the modules.

The remainder of this paper is organized as follows: Section 2 introduces related works. In Section 3, the proposed framework for the classification task is presented. In Section 4, experiments with a clinical dataset are performed to evaluate the proposed method. The dataset information, implementation details, and evaluation metrics are also illustrated. In Section 5 and Section 6, the experimental results and discussion are presented. Conclusions are drawn in Section 7.

## 2. Related Works

### 2.1. Deep Learning in MRI Analysis

Great progress has been made in deep learning-based methods for several MRI-related tasks. For segmentation, Smarta et al. [25] designed a modified U-Net architecture under a deep learning framework for the detection and segmentation of brain tumors from MR images. For classification, EL-Geneedy et al. [18] proposed an analysis pipeline utilizing a shallow convolution neural network to make a fast and accurate Alzheimer’s disease diagnosis; Jyotismita et al. [19] designed the Brain Tumor Segmentation and Classification Network to properly classify three types of brain tumors from MR images. For MRI reconstruction, Wu et al. [26] used the Swin Transformer as the backbone to restore high-quality MRI images from undersampled k-space data in an end-to-end manner; Guo et al. [27] proposed a joint group sparsity-based network for multi-contrast MRI reconstruction, enhancing the reconstruction efficiency and accuracy by processing a joint sparsity algorithm. The successes achieved by deep learning-based models inspired us to incorporate this technology into the task of MRI-based ACL tear detection.

### 2.2. Attention

Attention is an important mechanism employed in deep learning methodologies and is prominently featured in both Convolutional Neural Networks (CNNs) and Transformer models. It enables the system to selectively focus on different parts of the input, thereby enhancing its ability to capture salient information and context, leading to improved performance and interpretability. Initially, Dzmitry et al. [21] used attention to allow a model to automatically (soft-)search for parts that are relevant to predicting a target. Hu et al. [23] proposed the Squeeze-and-Excitation block to adaptively recalibrate channel-wise feature responses by explicitly modeling interdependencies between channels. Sanghyun et al. [28] sequentially inferred attention maps along both channel and spatial dimensions for adaptive feature refinement. Ashish et al. [29] proposed the Transformer architecture using a multi-head self-attention mechanism, which has been one of the most commonly used architectures in deep learning fields. The multi-head self-attention module was then introduced to the image process by Alexey et al. [30]. However, unlike natural images, medical images, such as MRI scans, possess a unique structure typically presented as a 3D volume. The contextual information spanning various depths or layers holds significant importance for medical image analysis tasks. Inspired by insights from previous research emphasizing attention mechanisms, we explored cross-layer correlations to enhance model performance by incorporating an attention module.

## 3. Method

**Figure 1:**
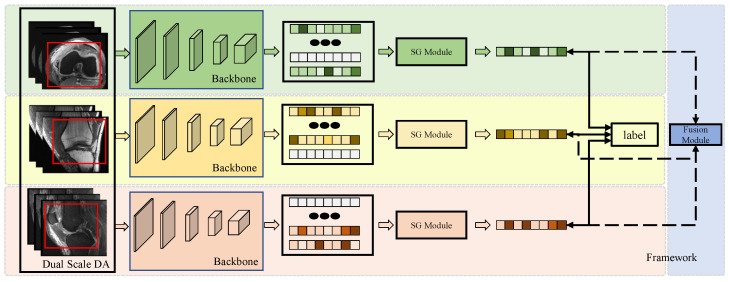
The framework of the model. Each individual perspective of the MRI scan is augmented using the Dual-Scale Data Augmentation module (Dual-Scale DA) first and then fed into the backbone to extract feature representations. The red box presents the randomly selected area in the step1 of Dual-Scale DA module. Then, the features are passed through the Selective Group Attention Module (SG Module). The aggregation features can be used to make predictions directly and fused in the Fusion Module to make the final classification.

The framework of the model is illustrated in Figure 1. The proposed model consists of three main modules: (1) the Dual-Scale Data Augmentation module, (2) the selective group attention module, and (3) the fusion module. The scan image of each individual perspective is processed with dual-scale data augmentation and fed into the basic backbone to generate the original feature map. Then, the selective group attention module is used to capture relationships across the layer, channel, and space scales. Finally, the fusion module assembles the predictions made with different input views to obtain the final classification result. Details of the proposed model are discussed in the following subsections.

### 3.1. Dual-Scale Data Augmentation

**Figure 2:**
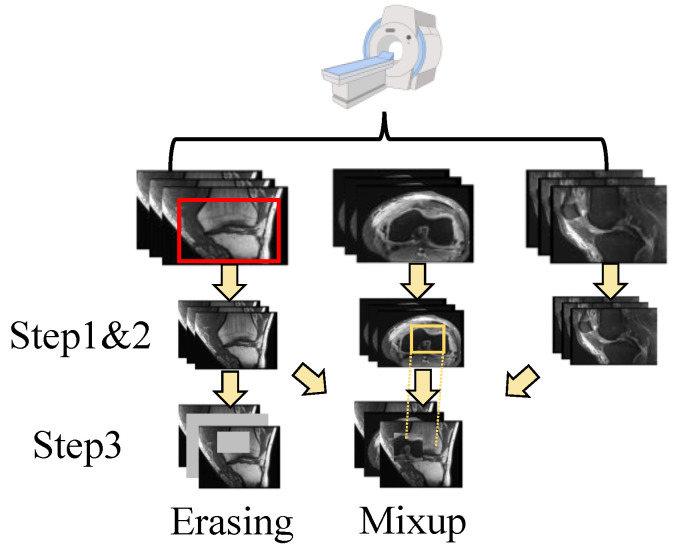
The pipeline of data processing. After scanning, there are three perspectives of the images. For each individual perspective, there are three steps for dual-scale data augmentation as follows: step 1. random crop; step 2. normalization; and step 3. erasing or mixup. The image is randomly cropped from the original image (as shown in the top row, where the red box is the cropped area). Then, the image is normalized with (1). Next, the image is augmented using a multi-scale erasing or mixup strategy. The patches chosen for the mix-up strategy are selected from other views through random selection (as shown in yellow box). More details can be found in Section 3.1.

The shortage of data has been a persistent challenge in medical image analysis. Data augmentation offers a promising solution to mitigate this limitation. In contrast to techniques commonly applied to natural images, we modified two methods, erasing and mixup, to suit the specific structure of the data used in this study. In addition, we also use random crops as another augmentation method. The augmentation operation is integrated into the data processing flow used in MRNet [15]. The details are shown in Figure 2.

First, for each input dataset with a shape of L×256×256 (*L* denotes the layer count), it is randomly cropped from 256 × 256 to 224 × 224. Random cropping is a data-augmentation technique that involves extracting a random subset from an original image. This enhances the model’s generalization capabilities because the objects of interest may not always be fully visible or present at the same scale in training data. Then, the cropped image is normalized with the statistics calculated using training data. For each pixel $p_{l,i,j}$ of the image, the value is changed to $p_{l,i,j}^{\prime }$ with:

(1) $$p_{l,i,j}^{\prime }=\frac{p_{l,i,j}-m}{std},\;l=1,\ldots L,\;i,j=1,\ldots ,224,$$

where *m* and std refer to the mean and standard deviation of the data, respectively. Standardizing the data facilitates better feature learning by the model. Next, two augmentation approaches are applied to the data: erasing and mixup. The augmentations are performed on both the spatial scale and layer scale. For the spatial augmentation strategy, the erasing/mixup center is randomly selected to ensure that the target area has the size of the input size × ratio. For the erasing approach, the target area is then set to zero. For the mixup approach, the target area is then mixed up with the area randomly chosen from other layers via the same strategy, and for layer-scale, a certain number of layers is randomly selected from all layers according to a specified ratio for processing. The layers chosen for the mix-up strategy are selected from other views through random selection. These operations offer a distinct advantage by enhancing the diversity of the data while preserving the class information. Random selection is used under Beta distribution.

### 3.2. Selective Group Attention Module

#### 3.2.1. Group Module

**Figure 3:**
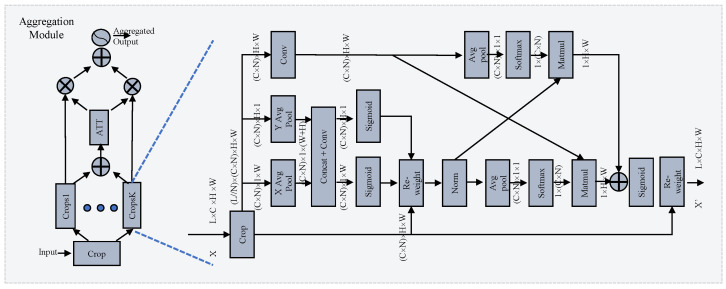
Illustration of the selective group attention module. On the left is the selective attention module, which carries out layer selection via the attention mechanism. On the right is the group module, which establishes the relationship across layers. More details can be found in Section 3.2.

Inspired by EMA [31], we propose a novel cross-layer learning method for establishing both short- and long-range dependencies on layer-scale. The details are shown in the right part of Figure 3. The output of the backbone $X\in R^{B\times L\times C\times H\times W}$ is the input of this module. It is divided into N crops [$C_{1},C_{2},\ldots C_{N}$], where the shape of each sub-crop is B×L//n×(C×n)×H×W. B, L, C, H, and W denote the batch size, layer count, channel count, height, and width of the feature map, respectively. For clarity, later the shape is referred to as L//n×(C×n)×H×W, omitting the *B*. This operation forces the module to investigate the relationship across layers along the channel dimension. The EMA-like structure utilizes three parallel pathways to extract attention weights from the grouped feature maps. Two distinct branches capture long-range interactions spatially along the vertical and horizontal directions with specific 1-D average pooling layers:

(2) $${C_{n}}_{H}={\mathrm{AvgPool}}_{H}\left(C_{n}\right),{C_{n}}_{W}={\mathrm{AvgPool}}_{W}\left(C_{n}\right),\;n=1,\ldots N.$$

The attention map learned from two branches is aggregated with the original feature map, which preserves precise positional information and effectively exploits long-range dependencies:

(3) $$\;\;\;\;\;\;\;\;\;\;\;\;\;\;\;\;\;\;\;A=\mathrm{Conv}\left(\left[{C_{n}}_{H},{C_{n}}_{W}\right]\right)\in R^{L//N\times (C\times N)\times 1\times (H+W)},$$

(4) $$A_{H},A_{W}=A,\;\;\;\;\;\;\;\;\;\;\;\;\;\;\;\;\;\;\;\;\;\;\;\;\;\;\;\;\;\;\;\;\;\;\;\;\;\;\;\;\;\;\;\;\;\;\;\;\;\;\;\;\;\;\;\;\;\;\;\;\;$$

(5) $$A_{H}^{\prime }=\mathrm{Sigmoid}\left(A_{H}\right)\in R^{L//N\times (C\times N)\times H\times 1},$$

(6) $$\;A_{W}^{\prime }=\mathrm{Sigmoid}\left(A_{W}\right)\in R^{L//N\times (C\times N)\times 1\times W},$$

(7) $$A^{\prime }=A_{H}^{\prime }⊗A_{W}^{\prime }\in R^{L//N\times (C\times N)\times H\times W},\;\;\;\;$$

where the permutation operation for multiplication is omitted and ⊗ denotes matrix multiplication. Formula (4) denotes the separation of *A* along the vertical and horizontal directions. The third branch captures the global information using a combination of a convolution layer and a 2-D averaging pooling layer. Finally, the fusion of context information with global and local information allows the module to generate more refined attention for feature maps.

#### 3.2.2. Selective Attention Module

Investigating the relationship among crop groups across different channel and spatial scales enhances the generation of more focused attention maps for features. However, the number of layers integrated can significantly impact diagnostic performance. Similar to how medical experts pinpoint target areas in scan images, our goal is to ensure that attention maps concentrate effectively on the regions of interest. To address this challenge, we propose a novel selective attention module for layer selection, as illustrated in the left-hand side of Figure 3. The original feature map is divided into K crop groups and the attention is calculated as follows:

(8) $$\mathrm{Crop}\;\mathrm{Group}=[{\mathrm{crops}}_{1},{\mathrm{crops}}_{2},\ldots ,{\mathrm{crops}}_{K}],$$

(9) $${\mathrm{crops}}_{k}={\mathrm{aggregate}}_{k}\left(\mathrm{input}\right),$$

where the aggregation operation denotes the group module, which adopts a different layer size for each group, and “input” denotes the features generated by the backbone. $\left[F_{1},F_{2},\ldots ,F_{K}\right]$ denotes the enhanced feature maps with different aggregations. The enhanced feature maps are fed into the ATT block, as shown in Figure 3, to calculate the attention. First, the outcomes of the individual crop groups $F^{\prime }$ are fused via element-wise summation:

(10) $$F^{\prime }=F_{1}+F_{2}\ldots +F_{K}.$$

Then, a 2-D averaging pooling layer is employed to extract the global information. A compact feature $F^{\prime \prime }$ is created to enable guidance for adaptive selections with a group of fully-connected layers (FCs), and the final selection is made by the softmax function (Softmax):

(11) $$\;\;\;\;\;\;\;\;\;\;\;\;\;\;\;\;\;\;\;\;\;z=\mathrm{Softmax}(\mathrm{FC}\left(F^{\prime \prime }\right)),$$

(12) $$\mathrm{output}=\mathrm{sum}(z*F^{\prime \prime }),$$

where *z* denotes the attention weights and the weighted summation aims to select the best crop group.

### 3.3. Fusion Module

For each individual view, the output of the selective group attention module (SG module) can be used for final prediction with fully connected layers. However, just as radiologists often rotate scan images to find more information, we utilize a fusion module to explore the inter-relationships among various perspectives to achieve the final classification. There are many methods for investigating the fusion of results from different models fed with the same input. Here, we fuse the models’ outcomes fed with different views according to the fuzzy distance [32]. Let $S_{a}\left(x\right),S_{c}\left(x\right),\mathrm{and}\;S_{s}\left(x\right)$ be the confidence scores of sample x assigned by the model fed with axial, coronal, and sagittal views, respectively. For each sample x, and class label *j*, the distance (*P*) between the ideal solution vector **1**, and the confidence score can be calculated as follows:

(13) $$P_{j}=\left(1-S_{a}^{j}\left(x\right),1-S_{c}^{j}\left(x\right),1-S_{s}^{j}\left(x\right)\right).$$

In this study, the Euclidean, Manhattan, and cosine distances are used for the ensemble. The information from these three measures is combined with the product for each class as follows:

(14) $$I_{j}\left(x\right)=P_{j}^{E}\left(x\right)\times P_{j}^{M}\left(x\right)\times P_{j}^{C}\left(x\right),$$

where $P_{j}^{E}$, $P_{j}^{M}$, and $P_{j}^{C}$ stand for the Euclidean, Manhattan, and cosine distances, respectively. The final prediction is made with the following:

(15) $$y=argmin\{I_{j}\left(x\right)\},$$

where *y* is the class label assigned to sample **x**.

## 4. Experiment

### 4.1. Data Preparation

In this study, we used a public dataset called MRNet [15], which was collected at Stanford University Medical Center. Examinations were performed with GE scanners (GE Discovery, GE Healthcare, Waukesha, WI) with a standard knee MRI coil and a routine non-contrast knee MRI protocol. For each case, three views of the data were obtained: axial, coronal, and sagittal. Here, the case with an ACL tear was defined as positive. The details of the dataset are shown in Table 1.

### 4.2. Implementation

In this study, we employed PyTorch to implement all methodologies on an Ubuntu 18.04 server featuring Nvidia Tesla-V100 graphics processing units (GPUs). All experiments were conducted on the training and validation sets of the dataset. The training set was divided into training and tuning subsets at a ratio of 0.8:0.2 for five-fold cross-validation. To make a substantial and fair comparison, the dataset was split using a stratified k-fold sampling strategy to maintain the class distribution. We trained the models using a consistent setting. The initial learning rate was set to $1\times 10^{-5}$ and reduced by a factor of 0.8 when the validation loss stopped improving for five epochs. The optimizer used in this study was Adam, with a weight decay of 0.01. A weighted loss function was used to calculate the model performance, and further details of the loss function are discussed in the Discussion Section 6. All the proposed modules were designed to be easily adaptable to the original backbone. In this study, the backbone employed was MRNet.

### 4.3. Metrics

To assess the performance of various models, we utilized accuracy, sensitivity, specificity, and AUC metrics to qualitatively compare their effectiveness. The sensitivity and specificity were calculated and defined by viewing the normal case as negative and an existing ACL tear as positive.

## 5. Results

In this section, the experimental results are listed. First, we compared the performances of different models, including the proposed SGNETmodel, and the MRNet [15], DLD [33], ELNet [34], VIT [35], and Med3D [36] models. MRNet uses AlexNet as its backbone. DLD is a deep learning-based ACL tear detector that uses cascade models to locate objects and perform classification. [34] proposed multi-slice normalization and BlurPool operations to enhance model performance. Then, we replaced the backbone of the MRNet with VIT and Med3D. The former was pretrained on the ImageNet22k [37] natural image dataset, while the latter was pretrained on medical images. Then, we conducted a wide range of experiments to verify the effectiveness of the proposed modules. In addition, we performed an ablation study on the parameter settings. The details are outlined in the subsequent subsections.

### 5.1. ACL Classification

The quantitative results obtained by different approaches are shown in Table 2. The proposed method, SGNET, achieved the best results among most metrics, except for specificity. MRNet achieved the best specificity. This discrepancy may be attributed to the loss weight configuration during training, as evidenced by MRNet’s poor sensitivity performance of 0.7590. Our proposed model aims to achieve optimal performance in both positive and negative case classifications. The inferior performance of the VIT model compared to that of other approaches could be attributed to insufficient training data. Visual transformers typically require a large amount of data for effective training. Replacing the backbone with Med3D resulted in a significant improvement in performance, suggesting that pretraining with medical images is beneficial.

### 5.2. Module Investigation

**Figure 4:**
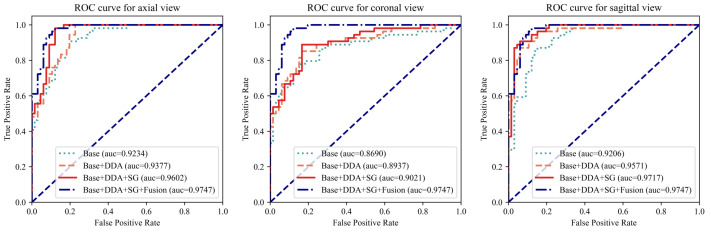
The ROC curves for each individual view with different model settings. The left, middle, and right figures plot the curves for the axial, coronal, and sagittal views, respectively. For each view, the ROC curve of the complete model (Base + DDA + SG + Fusion) was added for comparison. “Base” refers to using only an MRNet-like backbone. “DDA” represents the use of the dual-scale data augmentation strategy. “SG” and “Fusion” denote the leverage of the selective group attention module and the fusion module, respectively.

To comprehensively evaluate the proposed modules, an extensive array of experiments was conducted, encompassing all three views. As depicted in Figure 4 and Table 3, the proposed modules resulted in significant improvements in model performance when fed with each individual view and further enhanced performance through the fusion of three views. Taking the AUC as the evaluation metric, which balances the classification accuracy across positive and negative cases while mitigating the impact of class imbalance, the augmentation techniques yielded improvements of 1.4%, 2.4%, and 3.7% for the axial, coronal, and sagittal views, respectively. Furthermore, the integration of the SG module into the architecture demonstrated notable enhancements in performance, with 2.3%, 0.8%, and 1.5% improvements on the three views, respectively. Finally, the fusion module assembles the outcomes of three individual views into the final prediction, achieving the best differentiation performance with an AUC of 0.9747. As shown in Table 3, for all three views, the model with the proposed AUG and SG modules achieved the best accuracy. The fusion module further improved the performance. Even compared with the best results of the individual view, the fusion module enabled the proposed model to achieve an improvement of 2.5% in accuracy and 1.7% in sensitivity, with only a 0.17% decrease in specificity compared to a highly unbalanced result in the axial view. The results of these experiments validate the effectiveness of the proposed modules.

### 5.3. Other Ablation Study

First, we conducted ablation studies on different data augmentation strategies. Here, we used the sagittal view as input. The outcomes were obtained before the fusion modules. The strategies we tested included the following: 1. erasing; 2. mixup; 3. both combined. We tested the different parameter settings of the former two approaches and then combined the best two approaches. The results are shown in Table 4. The erasing strategy achieved the best results with a ratio of 0.5, while the mixup strategy obtained the best results with ratio of 0.25. The combination strategy yielded poor performance. One explanation for this phenomenon is that over-augmentation leads to the loss of crucial information in the input, thereby adversely affecting the classification performance. According to the results of the ablation study, a mixup strategy with a ratio of 0.25 and an erasing strategy with a ratio of 0.5 were employed during training.

## 6. Discussion

### 6.1. ACL Diagnosis

Making an accurate diagnosis based on MRI is a crucial approach in the clinical diagnosis of ACL tears, and it relies heavily on the experience of clinicians. Deep learning methods, which rely on annotated images, can automatically learn the features related to the classification of MRI and achieve a good performance on this task, thereby assisting clinicians in making diagnoses. In Section 5.1, the results of the proposed model, SGNET, and other deep learning-based models are listed in Table 2. MRNet was developed based on this eponymous dataset; DLD is one of the state-of-the-art methods that employ artificial intelligence techniques for this task; and ELNet is a modified classic convolution network that incorporates multi-slice normalization along with BlurPool downsampling to enhance diagnostic performance. The experimental results shown in Table 2 demonstrate that, compared with these state-of-the-art methods, the proposed method still achieved the best performance among the comparisons. Therefore, we believe that this deep learning network can be considered an effective tool for clinical application in ACL tear detection. The proposed model performs well on both positive and negative cases, leading to higher overall accuracy rather than achieving extremely high results on only one metric.

Notably, in the original paper of MRNet [15], the model demonstrated high specificity for ACL tear detection, with a specificity of 0.9680, but achieved a relatively low sensitivity of 0.7590. While this high specificity is advantageous for reducing the rate of false-positive diagnoses, which is clinically significant, it also highlights a weakness stemming from the unbalanced distribution of data classes. In this study, we trained the model with focal-loss [38] ($L_{fl}$) to mitigate this problem in addition to the simple weighted loss ($L_{wl}$) that is employed in MRNet. Mathematically, the total loss (L) is defined as follows:

(16) $$L=L_{wl}+L_{fl},$$

(17) $$L_{wl}=-\alpha_{t}log\left(p_{t}\right),$$

(18) $$\;\;\;\;\;\;\;\;\;\;\;\;\;\;\;\;\;L_{fl}=-\alpha_{t}{\left(1-p_{t}\right)}^{\gamma }log\left(p_{t}\right),$$

(19) $$\;\;\;\;\;\;\;\;\;\;\;\;\;\;\;\;\;\;\;\;\;\;\;p_{t}=\left\{\begin{array}{c} p\;\;if\;y=1,\\ 1-p\;\mathrm{otherwise}, \end{array}\right.$$

(20) $$\;\;\;\;\;\;\;\;\;\;\;\;\;\;\;\;\;\;\;\;\;\;\;a_{t}=\left\{\begin{array}{c} a\;\;if\;y=1,\\ 1-a\;\mathrm{otherwise}, \end{array}\right.$$

where α is the weight factor for class balancing, p denotes the estimated probability for the class with label *y* = 1, and γ is the modulating factor. We reproduced MRNet under the conditions mentioned above, which are identical to those of the proposed model, yielding an accuracy of 0.8750, a sensitivity of 0.8636, a specificity of 0.8889, and an AUC of 0.9497. These experimental findings imply a substantial potential for enhancing the performance of artificial intelligence-based automated diagnostic techniques. Techniques such as focal loss, which address the problem of class imbalance, are highly needed not only in medical image processing but also in natural image analysis. This area is worthy of further exploration.

Additionally, unlike MRNet and the proposed model, which uses the whole image as input, DLD [33] employs cascade models for this task: the first model locates the ACL and the second model classifies the ACL as normal or torn. Medical images are highly complex and filled with intricate details, posing a challenge for models in terms of extracting relevant features. By employing a detection model that narrows the region of interest and filters out superfluous information, it enhances feature extraction and results in better classification performance in the next step. Thus, the creation of an effective detection model is anticipated to further bolster the performance of the proposed model. We intend to undertake relevant research in future work.

### 6.2. External Test

To verify the robustness of the proposed model, which has important clinical applications, we conducted an external test on the KneeMRI dataset [39]. This is also a public dataset. The MR data were retrospectively gathered at the Clinical Hospital Centre in Rijeka, Croatia, from 2006 until 2014. The type of ACL injury was established in a double-blind fashion by comparing the retrospectively set diagnosis against the prospective opinion of another radiologist. After clean-up, the resulting dataset consisted of 917 usable, labelled exam sequences of left or right knees. The dataset only consists of sagittal examinations, so the fusion model of the proposed model used a single input in this experiment. The details of the dataset are shown in Table 5.

In this experiment, the partially- and completely-injured cases were considered positive cases, while the non-injured cases were referred to as negative cases. Without retraining our model on the external dataset, it achieved an AUC of 0.8657, which is better than the result (AUC = 0.86) mentioned in [33]. The results are lower than those from the internal test, suggesting significant potential for improving model robustness. To enhance performance, in addition to retraining on the new dataset directly, several techniques are planned for future work. Domain adaptation could be an effective solution for addressing the gap between the two datasets. Additionally, leveraging larger models and larger datasets is currently a popular approach for improving the generalizability of models.

### 6.3. Data

Aside from the previously mentioned weakness of data class imbalance, the impact of the dataset is also evident in other experimental outcomes. Table 4 reflects the significance of data augmentation. When configured with suitable parameters, data augmentation strategies prove advantageous for model enhancement. Among the comparative methodologies, several basic techniques, such as rotation, shift, and flip, which are used on spatial scales, are also included in MRNet. We performed a comparative experiment using the basic methods or the proposed Dual-Scale Data Augmentation module. The models were fed with a sagittal view. As shown in Table 6, the proposed Dual-Scale Data Augmentation module was effective, surpassing the performance of models utilizing conventional augmentation techniques.

Additionally, as seen in Table 2, the VIT model failed to achieve good performance, potentially due to the substantial amount of training data required for a transformer-based model to converge. Nonetheless, even with a limited dataset, by pretraining the model on medical image datasets, Med3D obtains good performance compared to the MRNet. Here, we employed RESNet with a parameter count comparable to that of MRNet, which serves as the backbone architecture. This highlights the crucial role of model pretraining on task-related data, emphasizing its efficacy in boosting performance. Collecting and curating an extensive MRI dataset, especially on knee joint examinations, and appropriately pretraining models on such datasets are beneficial for improving model performance on relevant tasks, which will be considered in future studies.

According to the issues and findings mentioned above, in future work, we have identified several main directions. For data, the tasks include building a larger and more comprehensive dataset, and exploring methods to better leverage limited data. For the model, we plan to identify improved frameworks, such as adjusting the attention module, that can discover better task-related image representations to enhance classification performance.

## 7. Conclusions

In summary, this study presents the development and validation of a deep learning model, SGNET, designed to identify and predict anterior cruciate ligament (ACL) tears using magnetic resonance imaging (MRI). The model, which integrates a Dual-Scale Data Augmentation (DDA) module, a selective group attention (SG) module, and a fusion module, has demonstrated robust performance in detecting ACL tears across various segments of the knee on MR images. The recognition accuracy, specificity, and sensitivity metrics highlight its potential as a reliable diagnostic tool. The application of SGNET holds significant translational potential, as it may significantly reduce the misdiagnosis rate of ACL injuries and provide a valuable asset to clinicians by streamlining the diagnostic process.

## Figures and Tables

**Table 1:** The demographic information of the dataset.

Statistic	Training	Validation
Number of exams	1130	120
Number of patients	1088	111
Number of female patients (%)	480 (42.5)	50 (41.7)
Age, mean (SD)	38.3 (16.9)	36.3 (16.9)
Number with ACL tear (%)	208 (18.4)	54 (45.0)
Number w/o ACL tear (%)	922 (81.6)	66 (55.0)

**Table 2:** Comparison of model performance in terms of ACL classification.

Model	ACC	SEN	SPE	AUC
MRNet	0.8670	0.7590	**0.9680**	0.9650
DLD	0.8750	0.8500	0.8900	0.9620
ELNet	0.9000	0.9070	0.8940	0.9560
VIT	0.8500	0.8182	0.8889	0.9043
Med3D	0.8917	0.8788	0.9074	0.9290
SGNET	**0.9250**	**0.9259**	0.9242	**0.9747**

The best results of all experiments are highlighted in bold.

**Table 3:** Investigation of the efficacy of the modules.

View	Modules				ACC	SEN	SPE
	Base	DDA	SG	Fusion			
Axial	✓				0.8250	0.7424	**0.9259** *
	✓	✓			0.8333	0.8333	0.8333
	✓	✓	✓		0.8917 *	0.9091 *	0.8704
Coronal	✓				0.8083	0.8182	0.7963
	✓	✓			0.8333	0.8182	0.8519
	✓	✓	✓		0.8583 *	0.8333 *	0.8889 *
Sagittal	✓				0.8333	0.8030	0.8704
	✓	✓			0.8917	0.9091 *	0.8704
	✓	✓	✓		0.9000 *	0.8939	0.9074 *
All	✓	✓	✓	✓	**0.9250**	**0.9259**	0.9242

✓ denotes the use of the specific module. “Base” refers to using only the MRNet-like backbone. “DDA” represents the use of the dual-scale data augmentation strategy. “SG” and “Fusion” denote the leverage of the selective group attention module and the fusion module, respectively. The best results for each perspective are noted with “*”. The best results of all experiments are highlighted in bold.

**Table 4:** Comparison of different data augmentation strategies.

Strategy	ACC	SEN	SPE	AUC
Erasingrate = 0.25	0.8650	0.8606	0.8704	0.9447
Erasingrate = 0.50	0.8767	0.8545	**0.9037**	0.9594
Erasingrate = 0.75	0.8683	0.8485	0.8926	0.9403
Mixuprate = 0.25	**0.8933**	**0.8879**	0.9000	**0.9628**
Mixuprate = 0.50	0.8700	0.8697	0.8704	0.9498
Mixuprate = 0.75	0.8117	0.8424	0.7741	0.9044
Erasing + Mixup	0.8667	0.8788	0.8519	0.9400

The best results of all experiments are highlighted in bold.

**Table 5:** Data distribution of KneeMRI dataset.

KneeMRI	Not-Injured	Partially-Injured	Completely-Ruptured	Total
Count	690	172	55	917
Percentage (%)	75.25	18.75	6.00	100

**Table 6:** Comparison of different augmentation methods.

	ACC	SEN	SPE	AUC
Basic methods	0.8667	0.8636	0.8704	0.9234
DDA module	**0.8833**	**0.8788**	**0.8889**	**0.9405**

The best results of all experiments are highlighted in bold.

## Data Availability

The data presented in this study are available at MRNet dataset [15] from Stanford University Medical Center, USA, and KneeMRI dataset [39] from Clinical Hospital Centre Rijeka, Croatia.

## Abbreviations

The following abbreviations are used in this manuscript:

MRI	magnetic resonance imaging
ACL	anterior cruciate ligament
DDA	Dual-Scale Data Augmentation module
SG	selective group attention
GPU	graphics processing unit
ROC	receiver operating characteristic
AUC	area under the ROC Curve
TP	true positive sample
TN	true negative sample
FN	false negative sample
FP	false positive sample
